# P04 The pain word cloud project: development of a clinic prompt to support young people in conversations about pain

**DOI:** 10.1093/rap/rkac067.004

**Published:** 2022-09-28

**Authors:** Tilda Kierkegaard Holt, Zahra Baz, Suruthi Gnanenthiran, Lee R Rebecca, Lunt E Laura, McDonagh E Janet

**Affiliations:** Your Rheum, A national advisory group of the Barbara Ansell National Network for Adolescent Rheumatology, Manchester, United Kingdom; Your Rheum, A national advisory group of the Barbara Ansell National Network for Adolescent Rheumatology, Manchester, United Kingdom; Your Rheum, A national advisory group of the Barbara Ansell National Network for Adolescent Rheumatology, Manchester, United Kingdom; Centre for Epidemiology Versus Arthritis, Centre for Musculoskeletal Research, Manchester Academic Health Science Centre, University of Manchester, Manchester, United Kingdom; Royal Manchester Children’s Hospital, Manchester University Hospitals Trust, Manchester, United Kingdom; Centre for Epidemiology Versus Arthritis, Centre for Musculoskeletal Research, Manchester Academic Health Science Centre, University of Manchester, Manchester, United Kingdom; NIHR Manchester Biomedical Research Centre, Manchester University NHS Foundation Trust, Manchester, United Kingdom; Centre for Epidemiology Versus Arthritis, Centre for Musculoskeletal Research, Manchester Academic Health Science Centre, University of Manchester, Manchester, United Kingdom; NIHR Manchester Biomedical Research Centre, Manchester University NHS Foundation Trust, Manchester, United Kingdom

## Abstract

**Introduction/Background:**

Pain is a predominant symptom that young people often present with in paediatric rheumatology clinics. There are several challenges with respect to pain communication between young people and healthcare professionals (Lee RR 2020). At a virtual Your Rheum meeting, (Your Rheum is a national young person’s advisory group in rheumatology), a word cloud was developed as part of the discussion to start conversations around the development of a future pain research project. Afterwards, the young people suggested the word cloud would potentially be useful to start discussions about pain in clinic.

**Description/Method Aim:**

to determine acceptability of using a word cloud as a communication tool to prompt discussions with young people aged 11-24 years about pain in clinic. Healthcare professionals in the Barbara Ansell National Network for Adolescent Rheumatology BANNAR network were asked by Your Rheum to give out the word cloud form to consecutive young people with pain in their clinic. The following data was collected: words in the current word cloud (consisting of 35 words) which described their pain, additional words to add to the word cloud, perception regarding the usefulness of a word cloud to aid conversations about pain, using a four-point scale ranging from ‘not at all’ – ‘yes a lot’. The age and gender of each young person was also collected. The information gathered was then collated by Your Rheum members.

**Discussion/Results:**

Of 33 young people who completed the forms, 26 were female and the mean age was 15 years, range 9-22 years. Young people selected a mean of 13/35 words from the word cloud, range 2-27 (table 1). Of the 33 young people, 14 suggested additional words (median of 2, range 1-24). Table 2 highlights additional words that could be added to the word cloud. When asked if a word cloud would help young people talk to health professionals about their pain in clinic, the majority said it yes it would (19=yes-a lot, 8=yes-a little, 5=possibly, 0=not at all 1=missing data).

**Key learning points/Conclusion:**

The word cloud was acceptable to the participants in this study and demonstrated potential as a communication prompt from the young person’s perspective. Research is needed to determine what impact it has on pain communication between the young person and healthcare professional in the actual clinic visit.

**P04 Table 1:** List of words from the word cloud selected by 50%> of respondents

Word	Number of respondents
Annoying	18
Ache	18
Achey	27
Constant	18
Fluctuating	15
Frustrating	22
Stiff	24

**P04 Table 2:** List of additional words suggested by 2 > respondents

Word	Number of respondents
Agony / Agonising	4
Depressing	2
Enduring	2
Painful	2
Unbearable	2

